# Association of *PADI2* and *PADI4* polymorphisms in COVID-19 host severity and non-survival

**DOI:** 10.1016/j.heliyon.2024.e27997

**Published:** 2024-03-15

**Authors:** Ilse Adriana Gutiérrez-Pérez, Ivette Buendía-Roldán, Oscar Zaragoza-García, Gloria Pérez-Rubio, José Rafael Villafan-Bernal, Leslie Chávez-Galán, Isela Parra-Rojas, Rafael de Jesús Hernández-Zenteno, Ingrid Fricke-Galindo, Natividad Castro-Alarcón, Brandon Bautista-Becerril, Ramcés Falfán-Valencia, Iris Paola Guzmán-Guzmán

**Affiliations:** a Faculty of Chemical-Biological Sciences, Universidad Autónoma de Guerrero, Chilpancingo, Guerrero, 39000, Mexico; b Translational Research Laboratory on Aging and Pulmonary Fibrosis, Instituto Nacional de Enfermedades Respiratorias Ismael Cosio Villegas, Mexico City, 14080, Mexico; c HLA Laboratory, Instituto Nacional de Enfermedades Respiratorias Ismael Cosío Villegas, Mexico City, 14080, Mexico; d Investigador por Mexico, Laboratory of Immunogenomics and Metabolic Disease, Mexican National Institute of Genomic Medicine (INMEGEN), Mexico City, 14610, Mexico; e Laboratory of Integrative Immunology, Instituto Nacional de Enfermedades Respiratorias Ismael Cosio Villegas, Mexico City, 14080, Mexico; f COPD Clinic, Instituto Nacional de Enfermedades Respiratorias Ismael Cosío Villegas, Mexico City, 14080, Mexico

**Keywords:** Outcome, COVID-19, *PADI2*, *PADI4*, Polymorphisms, Inflammatory parameters

## Abstract

**Background:**

Enzymes of the peptidylarginine deiminase family (PADs) play a relevant role in the pathogenesis of COVID-19. However, the association of single nucleotide polymorphisms (SNPs) in their genes with COVID-19 severity and death is unknown.

**Methodology:**

We included 1045 patients who were diagnosed with COVID-19 between October 2020 and December 2021. All subjects were genotyped for *PADI2* (rs1005753 and rs2235926) and *PADI4* (rs11203366, rs11203367, and rs874881) SNPs by TaqMan assays and their associations with disease severity, death, and inflammatory biomarkers were evaluated.

**Results:**

291 patients presented had severe COVID-19 according to PaO_2_/FiO_2_, and 393 had a non-survival outcome. Carriers of the rs1005753 G/G genotype in the *PADI2* gene presented susceptibility for severe COVID-19, while the heterozygous carriers in rs11203366, rs11203367, and rs874881 of the *PADI4* gene showed risk of death. The GTACC haplotype in *PADI2*-*PADI4* was associated with susceptibility to severe COVID-19, while the GCACC haplotype was a protective factor. The GCGTG haplotype was associated with severe COVID-19 but as a protective haplotype for death. Finally, the GTACC haplotype was associated with platelet-to-lymphocyte ratio (PLR), the GCACC haplotype with neutrophil-to-hemoglobin and lymphocyte and the GCGTG haplotype as a protective factor for the elevation of procalcitonin, D-dimer, CRP, LCRP, NHL, SII, NLR, and PLR.

**Conclusions:**

Our results suggest that the haplotypic combination of GTACC and some individual genotypes of *PADI2* and *PADI4* contribute to the subjects' susceptibility for severity and death by COVID-19.

## Highlights

• SNPs rs1005753, rs874881, rs11203367, and rs11203366 of PADs genes are associated with COVID-19 severity.
• The GTACC haplotype combination is associated with susceptibility to severe COVID-19 and death.
• The haplotype GCGTG protects against inflammation and death from COVID-19.
• The combination of *PADI2* and *PADI4* SNPs exhibits a synergistic role in the COVID-19 severity.

## 1 Introduction

The genetic background of individuals contributes to the differential immune response during SARS-CoV-2 infection and possibly to the severity of symptoms and clinical outcomes. Although some sociodemographic, lifestyle, and morbidity characteristics are consistently related to the severity and death by COVID-19, the presence of single nucleotide polymorphisms (SNPs) in genes whose proteins are involved in antiviral defense, SARS-CoV-2 entry, replication, and host inflammatory response and their geographic distribution should be considered [[1], [2], [3], [4]].

In severe COVID-19, there is an increase in circulating monocytes and neutrophils and an infiltration into lung tissue [5]. Additionally, activation of neutrophils promotes the release of neutrophilic extracellular traps (NETs) and the release of antimicrobial factors such as neutrophil elastase and myeloperoxidase, contributing to viral defense [[6], [7], [8], [9]]. Furthermore, the formation of NETs is triggered by the action of the enzyme peptidyl arginine deiminase 4 (PAD4) and chromatin decondensation during infection by several viruses [10], including rhinovirus [11], respiratory syncytial virus [12], and SARS-CoV-2. Consequently, activating such biological processes contributes to hyperinflammation, tissue damage, thrombosis, and pulmonary fibrosis [8,9,[13], [14], [15]].

Lung biopsies from SARS-CoV-2-infected patients showed that *PADI2* and *PADI4* are involved in the antiviral response and that their mRNA levels are elevated [16]. Also, PADI4 mRNA levels are upregulated in leukocytes from subjects with severe COVID-19 compared to moderate COVID-19 and healthy controls [17]. In consequence, PAD enzymes have been proposed as targets to suppress human coronavirus infection [18].

Genes of the PADs enzyme family (*PADI1, PADI2, PADI3, PADI4,* and *PADI6*) are in a single cluster region of approximately 334.7 kb on the short arm of chromosome 1 (1p36.1) [19]. All *PADI* genes share significant identity at the level of their coding nucleotide sequences; however, the mechanisms in which they participate are different [20]. The *PADI2* and *PADI4* genes are highly expressed in hematopoietic cells [21], and previous studies have shown that some SNPs in these genes are associated with autoimmune diseases. In rheumatoid arthritis (RA), the GTG haplotype [SNPs 89G/A (rs11203366), 90T/C (rs11203367), and 92G/C (rs874881)] of the *PADI4* gene confers susceptibility for presenting the disease, and higher levels of *PADI4* mRNA [[22], [23], [24], [25]]. Although no linkage disequilibrium exists between the SNPs of the *PADI2* and *PADI4* genes [26], it is suggested that the susceptibility for RA could be attributed to the presence of haplotypes in both genes and not only to the presence of individual variants [27]. Therefore, this study aims to analyze the individual association of SNPs and haplotypic combination in *PADI2* and *PADI4* with inflammation markers, severity, and death by COVID-19 in a Mexican population.

## 2 Materials and methods

### 2.1 | Ethical considerations, human samples and experimental design

The protocol for this cross-sectional study was approved by the local Research Ethics Committee and complied with the Helsinki Declaration (approval number C53-20). All patients agreed to participate and gave their written informed consent. The inclusion criteria were: men and women ≥18 years old hospitalized by SARS-CoV-2 infection at the National Institute of Respiratory Diseases Ismael Cosío Villegas in Mexico City from October 1st, 2020, to December 21st, 2021. All cases were confirmed by reverse transcriptase-polymerase chain reaction (RT-PCR) from nasopharyngeal swabs.

### 2.2 | Measurement of sociodemographic, clinical, and inflammatory markers

Data on sociodemographic and significant medical history, clinical were obtained from each patient's electronic record. Disease severity, invasive mechanical ventilation (IMV), and survival or death were recorded during the hospital stay. The severity of Acute Respiratory Distress Syndrome (ARDS) induced by COVID-19 was defined according to the classification of Villar and colleagues as: mild (PaO2/FiO2 > 200), moderate (PaO2/FiO2 101–200), and severe (PaO2/FiO2 ≤ 100) [28]. Death's outcome was considered non-survival and discharge due to improvement as survival.

The systemic inflammation indices and hematological and biochemical parameters were measured through blood samples obtained from the patients during the hospital admission employing automated analyzers. In a study recently published by our working group, seven leading indices of systemic inflammation (PLR, NLR, MLR, dNLR, SII, SIRI, and NHL) were reported as predictors of severity, the need for IMV, and death due to COVID-19 [29]. Therefore, they were analyzed in relation to the genotypes and haplotypes of the polymorphisms described in this study. Other inflammation indices that have been reported to be associated with the severity of COVID-19 consistently in other populations around the world were also evaluated, such as the Lymphocyte to C-Reactive Protein ratio (LCRP), serum levels of C-reactive protein (CRP), fibrinogen, D-dimer, and procalcitonin. For these last five parameters, we employed the cut-off values estimated as the best for our population according to the sensitivity and specificity of the ROC curves (Supplementary Table 1).

### 2.3 | Genotyping

Genomic DNA was isolated by standard techniques from a blood sample collected in tubes with EDTA as an anticoagulant. The SNPs were genotyped through allele discrimination assay using commercial TaqMan assays (Applied Biosystems, San Francisco, CA, USA). The evaluated *PADI2* SNPs were C_2190445_20 (rs1005753/Intron, cat. 4351379) and C_2190476_1_ (rs2235926/3′UTR, Cat.4351379). The evaluated *PADI4* SNPs were C_22275072_10 (rs11203366/Intron, cat: 4351379), C_22275081_10 (rs11203367/Intron, cat:4351379), and C_2995365_20 (rs874881/5′UTR, cat:4351379). We used quantitative polymerase chain reaction (qPCR) according to the supplier's instructions [StepOnePlus™, Applied Biosystems, Carlsbad, CA, USA]. The thermal cycling settings were: denaturation at 60 °C for 30 s, followed by 40 cycles of 95 °C for 10 min and 95 °C for 15-sec alignment and extension at 60 °C 1 min and 4 °C. Genotype analysis was performed using TaqMan Genotype software (Applied Biosystems™ Real-Time PCR system, USA). We extracted information on the frequency of each SNP in other populations from the ALFA project for comparison with our results.

### 2.4 *In silico* analysis for intronic variants

To gain insight into the impact of non-coding variants on gene splicing or expression, we performed an *in silico* analysis of the two SNPs located at non-coding regions (rs1005753 and rs2235926) employing Ensembl prediction tool, SpliceAI, Berkeley Drosophila Genome Project (BDGP) and the regSNP-intron.

### 2.5 | Statistical analysis

We used Stata v. 14.0 (StataCorp, College Station, TX, USA) and GraphPad Prism v.8.4 (GraphPad Software, San Diego, CA, USA) for Windows. The categorical variables were expressed as the numbers and proportions and were compared using the Chi-squared test. We estimated the median, percentiles (p5th-p95th), and the Kruskal Wallis test for quantitative variables. The predictive values of LCRP, CRP, fibrinogen, D-dimer, and procalcitonin were determined by analyzing an ROC (receiver operating characteristic) curve and the area under the curve (AUC) with its 95% confidence interval. In addition, the cut-off values were defined from the sensitivity and specificity of each inflammation parameter using the Youden index.

Allele and genotype frequencies of SNPs were calculated by direct counting, and the Hardy-Weinberg equilibrium (HWE) was estimated for each SNPs. The differences in the distributions of allele and genotype frequencies and the associations of these with clinical characteristics of COVID-19 were performed using the Chi-square test. For the outcome "severe COVID-19," the control group was mild-to-moderate COVID-19; for the outcome "Non-survival," the control group was formed by survivors. The association and pairwise measure of linkage disequilibrium of the SNPs for *PADI2* (rs1005753 and rs2235926) and *PADI4* (rs11203366, rs11203367, and rs874881) was calculated using SHEsis software [30]. The association of SNPs and haplotypes of *PADI2* and *PADI4* with inflammatory markers, severity, and non-survival was determined using a logistic regression model, determining odds ratios (OR) and 95% confidence intervals (CI 95%). Results were considered significant at *p* < 0.05.

## 3 Results

### 3.1 | Demographic information, clinical data, and inflammatory markers

Of 1045 included subjects, 186, 568, and 291 had mild, moderate, and severe ARDS (27.8%), respectively. The proportion of patients who required IMV and died was 82.6% and 37.6%, respectively. ARDS severity was related to older age (*p* = 0.001), history of chronic respiratory disease (*p* = 0.002), delayed hospitalization (*p* < 0.001), length of hospital stay (*p* < 0.001) and elevated levels of inflammation markers such as WBC, RDW, NLR, dNLR, SII, SIRI, NHL, LCRP, D-dimer, and procalcitonin (*p* < 0.05) (Table 1).

**Table 1:** COVID-19 patients characteristics according to PaO_2_/FiO_2_ ratio category.

Characteristics	Total *n* = 1045	Mild *n* = 186	Moderate *n* = 568	Severe *n* = 291	*p*-value
Sociodemographic					
Age (years old)^a^	59 (34–81)	55 (33–82)	59 (36–81)	62 (33–82)	0.001
Sex, *n* (%)^b^					0.793
Women	364 (34.8)	63 (33.9)	195 (34.3)	106 (36.4)	
Men	681 (65.2)	123 (66.1)	373 (65.7)	185 (63.6)	
Age category, *n* (%)^b^					0.029
<35 years old	53 (5.1)	13 (7.0)	22 (3.9)	18 (6.2)	
35–50 years old	229 (21.9)	52 (28.0)	128 (22.5)	49 (16.8)	
51–65 years old	421 (40.3)	73 (39.2)	228 (40.1)	120 (41.3)	
>65 years old	342 (32.7)	48 (25.8)	190 (33.5)	104 (35.7)	
Body mass index (kg/m^2^)^a^	29.0 (22.8–40.8)	28.3 (22.8–41.1)	28.9 (22.8–40)	29.7 (22.7–41.6)	0.177
Body mass index category, *n* (%)^b^					0.063
Normal weight	175 (16.7)	35 (19.2)	88 (15.2)	52 (17.9)	
Overweight	411 (39.3)	78 (40.4)	239 (42.3)	94 (32.6)	
Obesity	459 (44.0)	76 (40.4)	240 (42.5)	143 (49.5)	
Tobacco smoking, yes, *n* (%)^b^	307 (29.4)	54 (29.0)	162 (28.5)	91 (31.3)	0.700
Type 2 diabetes, yes, *n* (%)^b^	315 (30.2)	56 (30.1)	180 (31.7)	79 (27.2)	0.406
Hypertension, yes, *n* (%)^b^	385 (36.9)	61 (32.8)	226 (39.8)	98 (33.8)	0.101
PCRD, yes, *n* (%)^b^	74 (7.1)	17 (9.1)	26 (4.6)	31 (10.7)	0.002
Clinical characteristics					
Symptoms onset (days)^a^	10 (4–20)	9 (4–20)	9.5 (4–21)	10 (3–21)	0.031
Length of stay (days)^a^	22 (8–71)	18 (5–63.5)	22 (9–71)	25 (11–75)	<0.001
IMV, yes, *n* (%)^b^	863 (82.6)	109 (58.6)	483 (85.0)	271 (93.1)	<0.001
Outcome, *n* (%)^b^					<0.001
Survival	652 (62.4)	147 (79.0)	361 (63.6)	144 (49.5)	
Non-survival	393 (37.6)	39 (21.0)	207 (36.4)	147 (50.5)	
Inflammatory markers					
WBC, 10^3^/6mm^3a^	9.9 (5.05–19.0)	9.2 (4.4–18.9)	9.9 (4.8–19.3)	10.4 (5.5–19.1)	0.002
Erythrocytes, 10^6^/mm^3a^	4.4 (2.9–5.4)	4.5 (3.2–5.4)	4.4 (2.9–5.4)	4.3 (2.8–5.4)	<0.001
Hemoglobin, gr/dL^a^	13.4 (8.8–16.7)	13.9 (9.2–16.7)	13.4 (8.8–16.7)	13.0 (8.5–16.9)	<0.001
Platelets,10^3^/mm^3a^	268.7 (123–485)	274.5 (134–503)	271 (117–479.6)	254 (123–487)	0.217
RDW^a^	14.1 (12.9–18.3)	13.9 (12.7–16.4)	14.1 (12.9–18.3)	14.4 (12.9–18.8)	<0.001
PLR^a^	390.1 (120.8–1266.6)	364.1 (124.1–1140)	387 (132–1210)	408 (112.6–1380)	0.365
NLR^a^	12.7 (2.92–51.0)	9.9 (2.7–41.5)	12.7 (3.4–52)	14.5 (2.4–51.0)	<0.001
MLR^a^	0.75 (0.2–2.5)	0.66 (0.2–2.0)	0.75 (0.22–2.5)	0.76 (0.2–2.75)	0.122
dNLR^a^	6.81 (1.78–18.75)	5.57 (1.55–16.53)	6.63 (1.93–18.57)	7.84 (1.50–20.40)	0.001
SII^a^	3338.7 (641.6–13509)	2821.7 (546.5–11160.6)	3376.7 (748.2–13530)	3785.4 (576–14869.3)	0.005
SIRI^a^	6.3 (1.0–30.9)	5.39 (0.96–23.4)	6.1 (1.03–32.2)	7.1 (1.03–32.2)	0.004
NHL^a^	0.98 (0.22–3.88)	0.75 (1.9–3.49)	0.96 (0.26–3.87)	1.17 (0.20–4.05)	<0.001
LCRP^a^	612.4 (126.7–7299.2)	815.6 (170.8–10000)	584.7 (132.0–7017.5)	523.84 (100.4–10810.8)	0.015
CRP,(mg/dL)^a^	10.8 (1.25–30.66)	9.43 (1.1–24.92)	10.9 (1.65–31.4)	11.4 (0.8–32.57)	0.134
Fibrinogen,(mg/dL)^a^	648.5 (358–964)	646.5 (359–916)	653 (377–964)	626 (265–1025)	0.183
D-Dimer,(μg/mL)^a^	1.32 (0.26–21.5)	0.8 (0.2–7.06)	1.20 (0.26–17.0)	1.84 (0.42–39.6)	<0.001
Procalcitonin,(mg/dL)^a^	0.19 (0.03–4.07)	0.13 (0.02–2.62)	0.20 (0.03–3.79)	0.22 (0.03–6.66)	0.003

Abbreviations: CRP, C-reactive protein; dNLR, derived neutrophil to lymphocyte ratio; IMV, invasive mechanic ventilation; LCRP, lymphocyte to CRP ratio MLR, monocyte to lymphocyte ratio; NHL, neutrophil-to-hemoglobin and lymphocyte; NLR, neutrophil to lymphocyte ratio; PCRD, previous chronic respiratory disease; PLR, platelet to lymphocyte ratio; RDW, red blood cell distribution width; SII, systemic immune-inflammation index; SIRI, systemic inflammation response index. WBC, white blood cell count.

*p*-value <0.05 was considered statistically significant.

a Data are expressed as the median and percentiles 5th-95th, compared using Kruskal-Wallis test.

b Data are expressed as the n (%), compared using the Chi-square test.

### 3.2 | Frequency of *PADI2* and *PADI4* gene SNPs and haplotype in COVID-19 patients

**Fig. 1:**
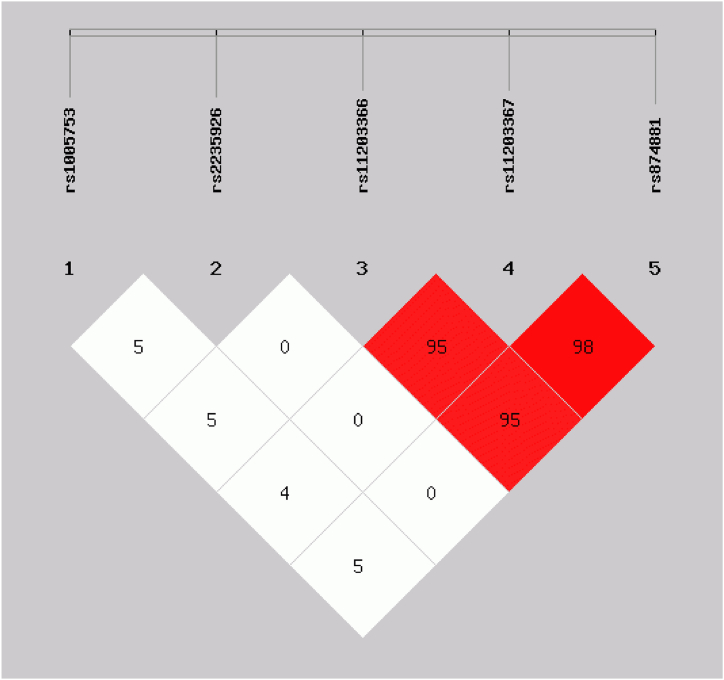
Linkage disequilibrium test of *PADI2* and *PADI4* polymorphisms. Haplotype frequencies and linkage disequilibrium (LD) were calculated using SHEsis software. Red area represents higher levels of LD. A D′ value of 100 indicates a complete LD between two markers and a D′ value of 0 indicates complete linkage equilibrium.

The allelic distribution of all *PADI2* and *PADI4* SNPs was in Hardy-Weinberg genetic equilibrium (*p* > 0.05). The haplotype analysis for *PADI2* SNPs identified two major haplotypes: TT (0.479) and TC (0.250), while for *PADI4* SNPs, the major haplotypes were GTG (0.525) and ACC (0.439). High linkage disequilibrium was observed for SNPs of the *PADI4* gene but not for *PADI2* SNPs neither for the interaction of *PADI2* and *PADI4* SNPs (Fig. 1). The haplotypic combination analysis between *PADI2* and *PADI4* SNPs discovered eight major haplotypes: TTGTG (0.249), TTACC (0.209), TCGTG (0.139), TCACC (0.102), GTGTG (0.088), GTACC (0.071), GCACC (0.057), and GCGTG (0.049) (Table 2). The frequencies of SNPs in other population's reveals that these variants are highly frequent in all populations, including ours, and that the frequencies of SNP's rs1005753 and rs2235926 in *PADI2* were the most different between populations (significant *p*-values).

**Table 2:** Genotypes, alleles and haplotypes frequency of *PADI2* and *PADI4* SNPs in COVID-19.

*PADI2* SNPs	*n* = 1045 (%)	HWE X^2^, *p*-value	Haplotypes of *PADI2*	*n* = 1045 (%)
rs1005753, *n* (%)		X^2^ = 1.43, *p* = 0.231	H1: 11/TT	1002 (0.479)
TT	548 (52.4)		H2: 12/TC	522 (0.250)
TG	428 (41.0)		H3: 21/GT	345 (0.165)
GG	69 (6.6)		H4: 22/GC	221 (0.106)
Allele, *n* (%)			Haplotypes of *PADI4*	
T	1524 (72.9)		H1: 111/GTG	1098 (0.525)
G	566 (27.1)		H2: 112/GTC	5 (0.002)
rs2235926, *n* (%)		X^2^ = 1.33, *p* = 0.247	H3: 121/GCG	7 (0.003)
TT	442 (42.3)		H4: 122/GCC	16 (0.008)
TC	462 (44.2)		H5: 211/ATG	23 (0.011)
CC	141 (13.5)		H6: 212/ATC	4 (0.002)
Allele, *n* (%)			H7: 221/ACG	19 (0.009)
T	1346 (64.4)		H8: 222/ACC	917 (0.439)
C	744 (35.6)		Haplotypic combination *PADI2* and *PADI4*	
*PADI4* SNPs			H1: 11111/TTGTG	521 (0.249)
rs11203366, *n* (%)		X^2^ = 1.45, *p* = 0.228	H2: 11222/TTACC	438 (0.209)
GG	313 (30.0)		H3: 12111/TCGTG	291 (0.139)
GA	500 (47.85)		H4: 12222/TCACC	214 (0.102)
AA	232 (22.2)		H5: 21111/GTGTG	184 (0.088)
Allele, *n* (%)			H6: 21222/GTACC	148 (0.071)
G	1126 (53.9)		H7: 22111/GCGTG	102 (0.049)
A	964 (46.1)		H8: 22222/GCACC	118 (0.057)
rs11203367, *n* (%)		X^2^ = 2.06, *p* = 0.151		
TT	317 (30.3)			
TC	496 (47.5)			
CC	232 (22.2)			
Allele, *n* (%)				
T	1130 (54.1)			
C	960 (45.9)			
rs874881, *n* (%)		X^2^ = 1.33, *p* = 0.247		
GG	324 (31.0)			
GC	499 (47.7)			
CC	222 (21.2)			
Allele, *n* (%)				
G	1147 (54.9)			
C	943 (45.1)			

Abbreviations: HWE, Hardy-Weinberg Equilibrium; SNPs, single nucleotide polymorphisms.Data *n* (%) using Chi-square test.

### 3.3 | *PADI2* and *PADI4* gene SNPs and the association with severity and non-survival in COVID-19 patients

The association analysis of individual SNPs indicated that the genotype related to susceptibility to severe COVID-19 was *PADI2* rs1005753 G/G (OR = 1.69, *p* = 0.045). Genotypes in *PADI4* related to a higher risk of death were the rs874881 G/C (OR = 1.37, *p* = 0.035), rs11203367 T/C, and rs11203366 G/A (OR = 1.44, *p* = 0.014). Moreover, the dominant model revealed that *PADI2* rs2235926 TC + CC was protective against death (OR = 0.77, *p* = 0.041), whereas the models TC + CC of rs11203367 (OR = 1.40, *p* = 0.016) and GC + CC of rs874881 (OR = 1.33, *p* = 0.040) of *PADI4* were associated with higher risk for death (Table 3).

**Table 3:** Association of *PADI2* and *PADI4* SNPs according to COVID-19 severity and non-survival.

*PADI2* SNPs	Mild or moderate *n=*754 (%)	Severe *n=*291 (%)	OR (95% CI), *p-*value	Survival *n=*652 (%)	Non-survival *n=*393 (%)	OR (95% CI), *p*-value
rs1005753, *n* (%)						
TT	397 (52.6)	151 (51.9)	1.0	340 (52.1)	208 (52.9)	1.0
TG	315 (41.8)	113 (38.8)	0.94 (0.70–1.26), 0.687	269 (41.3)	159 (40.5)	0.96 (0.73–1.26), 0.796
GG	42 (5.6)	27 (9.3)	1.69 (0.97–2.91), 0.045	43 (6.6)	26 (6.6)	0.98 (0.56–1.70), 0.964
Allele, *n* (%)						
T	1109 (73.5)	415 (71.3)	1.0	949 (72.8)	575 (73.2)	1.0
G	399 (26.5)	167 (28.7)	1.11 (0.89–1.39), 0.302	355 (27.2)	211 (26.8)	0.98 (0.79–1.20), 0.850
Dominant model, *n* (%)						
TT	397 (52.6)	151 (51.9)	1.0	340 (52.1)	208 (52.9)	1.0
TG + GG	357 (47.3)	140 (48.1)	1.03 (0.78–1.36), 0.82	312 (47.9)	185 (47.1)	0.96 (0.74–1.25), 0.807
rs2235926, *n* (%)						
TT	329 (43.6)	113 (38.8)	1.0	260 (39.9)	182 (46.3)	1.0
TC	322 (42.7)	140 (48.1)	1.26 (0.93–1.71), 0.112	299 (45.8)	163 (41.5)	0.78 (0.58–1.02), 0.068
CC	103 (13.7)	38 (13.1)	1.07 (0.67–1.67), 0.743	93 (14.3)	48 (12.2)	0.73 (0.48–1.11), 0.131
Allele, *n* (%)						
T	980 (65.0)	366 (62.9)	1.0	819 (62.8)	527 (67.0)	1.0
C	528 (35.0)	216 (37.1)	1.1 (0.89–1.34), 0.368	485 (37.2)	259 (33.0)	0.83 (0.68–1.0), 0.049
Dominant model, *n* (%)						
TT	329 (43.6)	113 (38.8)	1.0	260 (39.9)	182 (46.3)	1.0
TC + CC	425 (56.4)	178 (61.2)	1.21 (0.91–1.62), 0.159	392 (60.1)	211 (53.7)	0.77 (0.59–0.99), 0.041
*PADI4* SNPs						
rs11203366, *n* (%)						
GG	230 (30.5)	83 (28.5)	1.0	208 (31.9)	105 (26.7)	1.0
GA	349 (46.3)	151 (51.9)	1.19 (0.86–1.66), 0.259	299 (45.9)	201 (51.2)	1.33 (0.98–1.80), 0.056
AA	175 (23.2)	57 (19.6)	0.90 (0.59–1.35), 0.606	145 (22.2)	87 (22.1)	1.18 (0.82–1.72), 0.339
Allele, *n* (%)						
G	809 (53.7)	317 (54.5)	1.0	715 (54.8)	411 (52.3)	1.0
A	699 (46.3)	265 (45.5)	0.96 (0.79–1.17), 0.736	589 (45.2)	375 (47.7)	1.1 (0.92–1.32), 0.258
Dominant model, *n* (%)						
GG	230 (30.5)	83 (28.5)	1.0	208 (31.9)	105 (26.7)	1.0
GA + AA	524 (69.5)	208 (71.5)	1.09 (0.81–1.50), 0.530	444 (68.1)	288 (73.3)	1.28 (0.96–1.71), 0.076
rs11203367, *n* (%)						
TT	235 (31.2)	82 (28.2)	1.0	215 (33.0)	102 (25.9)	1.0
TC	347 (46.0)	149 (51.2)	1.23 (0.89–1.71), 0.198	294 (45.1)	202 (51.4)	1.44 (1.06–1.97), 0.014
CC	172 (22.8)	60 (20.6)	0.99 (0.66–1.49), 0.998	143 (21.9)	89 (22.7)	1.31 (0.90–1.89), 0.132
Allele, *n* (%)						
T	817 (54.2)	313 (53.8)	1.0	724 (55.5)	406 (51.7)	1.0
C	691 (45.8)	269 (46.2)	1.01 (0.83–1.23), 0.870	580 (44.5)	380 (48.3)	1.17 (0.97–1.40), 0.085
Dominant model, *n* (%)						
TT	235 (31.2)	82 (28.2)	1.0	215 (33.0)	102 (26.0)	1.0
TC + CC	519 (68.8)	209 (71.8)	1.15 (0.84–1.57), 0.346	437 (67.0)	291 (74.0)	1.40 (1.05–1.87), 0.016
rs874881, *n* (%)						
GG	243 (32.2)	81 (27.8)	1.0	217 (33.3)	107 (27.2)	1.0
GC	345 (45.8)	154 (52.9)	1.33 (0.97–1.86), 0.068	298 (45.7)	201 (51.2)	1.37 (1.01–1.85), 0.035
CC	166 (22.0)	56 (19.2)	1.01 (0.66–1.52), 0.952	137 (21.0)	85 (21.6)	1.25 (0.86–1.82), 0.205
Allele, *n* (%)						
G	831 (55.1)	316 (54.3)	1.0	732 (56.1)	415 (52.8)	1.0
C	677 (44.9)	266 (45.7)	1.03 (0.84–1.25), 0.738	572 (43.9)	371 (47.2)	1.14 (0.95–1.37), 0.137
Dominant model, *n* (%)						
GG	243 (32.2)	81 (27.8)	1	217 (33.3)	107 (27.2)	1.0
GC + CC	511 (67.8)	210 (72.2)	1.23 (0.91–1.68), 0.168	435 (66.7)	286 (72.8)	1.33 (1.0–1.77), 0.040

Abbreviations: CI, confidence interval; OR, odds ratio; SNPs, single nucleotide polymorphisms.1.0 Reference category. *p*-value <0.05.

### 3.4 | *PADI2* and *PADI4* gene SNPs and the association with inflammatory markers in COVID-19 patients

The association analysis of alleles and dominant genetic models with biomarkers of systemic inflammation revealed that the *PADI4* rs1005753 TG + GG model (OR = 1.33, 95%CI; 1.01–1.76, *p* = 0.028) and the G allele (OR = 1.23, 95%CI; 0.99–1.52, *p* = 0.048) are associated with the presence of a PLR ≥303 [Supplementary Table 2], as well as with values of LCRP ≤825 (OR = 2.48, 95%CI; 1.86–3.30, *p* < 0.001) and fibrinogen ≤687 (OR = 2.75, 95%CI; 1.70–4.48, *p* < 0.001) [Supplementary Table 3]. On the other hand, the *PADI2* rs2235926 TC + CC genetic model was found to be protective for D-dimer ≥1.25 μg/mL (OR = 0.73, 95%CI; 0.55–0.98, *p* = 0.031) [Supplementary Table 3]. However, we did not find an association between SNPs of *PADI4* and hematologic markers (Supplementary Table 4), but the A allele of rs11203366 (OR = 1.29, 95%CI; 1.03–1.63, *p* = 0.022), the C allele of rs11203367 (OR = 1.30, 95%CI; 1.03–1.63, *p* = 0.021), and the C allele of rs874881 (OR = 1.24, 95%CI; 0.98–1.56, *p* = 0.053) in the *PADI4* gene were associated with CRP values ≥ 9.9 mg/dL (Supplementary Table 5).

### 3.5 | Haplotypes and haplotypic combinations of *PADI2* and *PADI4* SNPs and their association with severity, non-survival, and inflammatory markers in COVID-19 patients

**Fig. 2:**
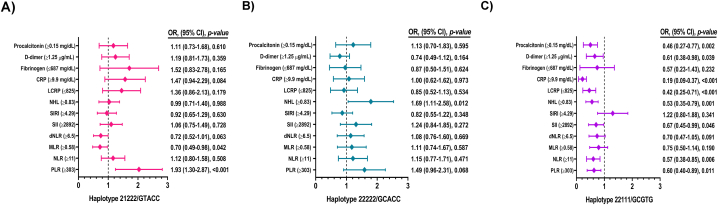
Association of the haplotypic combination of *PADI2* and *PADI4* polymorphisms with markers of inflammation in patients with COVID-19. The OR and 95% CI and *p* values were obtained by SHEsis test. *p*-value <0.05.

We found no association between individual *PADI2* and *PADI4* haplotypes and COVID-19 severity or death (Table 4). However, the haplotypic combination GTACC between SNPs of *PADI2* [rs1005753, rs2235926] and *PADI4* [rs11203366, rs11203367 and rs874881] was associated with susceptibility for severe COVID-19 (OR = 1.59, *p* = 0.007), as well as with susceptibility to present a PLR index ≥303 (OR = 1.93, *p* < 0.001) (Fig. 2A). Meanwhile, the GCACC combination was associated with the presence of NHL ≥0.83 (OR = 1.69, *p* = 0.012) (Fig. 2B) and with protection from severe COVID-19 (OR = 0.50, *p* = 0.005). On the other hand, the haplotypic combination GCGTG was associated with susceptibility for severe COVID-19 (OR = 2.07, *p* < 0.001) but as protective for elevated inflammation markers, including PLR ≥303, NLR ≥11, SII ≥2892, NHL ≥0.83, LCRP ≤825, CRP ≥9.9 mg/dL, D-dimer ≥1.25 μg/mL and procalcitonin ≥0.15 mg/dL (Fig. 2C).

**Table 4:** Haplotype frequencies and association with severity and non-survival according to *PADI2* and *PADI4* SNPs in COVID-19.

Haplotypes	PaO_2_/Fio_2_ >100 n = 754 (%)	PaO_2_/FiO_2_ ≤100 n = 291 (%)	OR (95% CI), *p*-value	Survival n = 652 (%)	Non-survival n = 393 (%)	OR (95% CI), *p*-value
***PADI2 SNPs***						
H1: 11/TT	737.6 (0.488)	264.18 (0.452)	0.868 (0.72–1.05), 0.148	612.36 (0.469)	390.12 (0.495)	1.11 (0.93–1.32), 0.235
H2: 12/TC	371.39 (0.246)	150.82 (0.258)	1.07 (0.86–1.33), 0.542	336.64 (0.258)	184.88 (0.235)	0.88 (0.72–1.09), 0.240
H3: 21/GT	243.39 (0.161)	107.82 (0.174)	1.10 (0.86–1.42), 0.454	207.64 (0.159)	136.88 (0.174)	1.11 (0.88–1.41), 0.373
H4: 22/GC	155.61 (0.103)	65.18 (0.112)	1.09 (0.80–1.49), 0.557	147.36 (0.113)	74.12 (0.094)	0.82 (0.61–1.09), 0.178
***PADI4 SNPs***						
H1: 111/GTG	789.63 (0.523)	307.94 (0.527)	0.99 (0.82–1.21), 0.980	702.79 (0.538)	394.77 (0.501)	0.88 (0.73–1.05), 0.178
H2: 112/GTC	4.13 (0.003)	1.02 (0.002)	ND	3.09 (0.002)	2.06 (0.003)	ND
H3: 121/GCG	4.95 (0.003)	1.94 (0.003)	ND	3.97 (0.003)	2.96 (0.004)	ND
H4: 122/GCC	10.29 (0.007)	6.10 (0.010)	ND	5.15 (0.004)	11.22 (0.014)	ND
H5: 211/ATG	20.29 (0.013)	3.04 (0.005)	ND	15.16 (0.012)	8.18 (0.010)	ND
H6: 212/ATC	2.95 (0.002)	1.0 (0.002)	ND	2.96 (0.002)	0.99 (0.001)	ND
H7: 221/ACG	16.13 (0.011)	3.08 (0.005)	ND	10.08 (0.008)	9.09 (0.012)	ND
H8: 222/ACC	659.63 (0.437)	257.88 (0.442)	1.0 (0.82–1.21), 0.980	560.8 (0.429)	356.74 (0.453)	1.13 (0.94–1.35), 0.178
**Haplotypic combination *PADI2* and *PADI4 SNPs***						
11111/TTGTG	379.93 (0.252)	138.95 (0.239)	0.91 (0.73–1.14), 0.445	334.78 (0.257)	189.48 (0.241)	0.93 (0.76–1.15), 0.528
11222/TTACC	328.39 (0.218)	114.89 (0.197)	0.87 (0.68–1.10), 0.251	264.86 (0.203)	169.48 (0.216)	1.09 (0.88–1.36), 0.399
12111/TCGTG	214.26 (0.142)	81.48 (0.140)	0.97 (0.73–1.27), 0.827	186.79 (0.143)	107.32 (0.137)	0.96 (0.74–1.24), 0.759
12222/TCACC	140.30 (0.093)	67.16 (0.115)	1.25 (0.92–1.71), 0.146	135.26 (0.104)	76.35 (0.097)	0.94 (0.70–1.26), 0.700
21111/GTGTG	137.95 (0.091)	42.89 (0.074)	0.78 (0.54–1.11) 0.171	111.48 (0.085)	70.84 (0.090)	1.07 (0.78–1.47), 0.647
21222/GTACC	93.68 (0.062)	56.12 (0.096)	1.59 (1.12–2.25), 0.007	85.82 (0.066)	66.59 (0.085)	1.33 (0.95–1.86), 0.089
22111/GCGTG	57.53 (0.038)	44.62 (0.077)	2.07 (1.38–3.10), <0.001	69.78 (0.054)	27.11 (0.034)	0.64 (0.40–1.00), 0.052
22222/GCACC	97.34 (0.065)	19.70 (0.034)	0.50 (0.30–0.82), 0.005	74.91 (0.057)	44.24 (0.056)	0.99 (0.67–1.45), 0.970

Abbreviations: CI, confidence interval; H, haplotype; ND, not determinate; OR, odds ratio; SNPs, single nucleotide polymorphisms.

The SNPs are listed in the order: *PADI2* rs1005753_T > G and rs2235926_T > C. *PADI4* rs11203366_G > A (89G/A), rs11203367_T > C (90T/C) and rs874881_G > C (92G/C).

The OR, 95% CI, and *p* values were obtained by SHEsis test.1.0 Reference category. *p*-value <0.05.

### 3.6 | *In silico* analysis of non-coding variants

The *Ensembl* variant effect predictor [VEP] revealed a potential impact of the rs2235926 on the splicing throughout the SpliceAI tool. Similarly, the BDGP *in silico* tool revealed that this SNP's fall within an acceptor site in the boundaries of intron-exon with a score of 0.95. However, we did not find any significant impact of the rs1005753 SNP on splicing after *in silico* analysis.

## 4 Discussion

Although the COVID Host Genomics Initiative (throughout a GWAS meta-analysis) revealed some SNP's (in genes *SFTPD, MUC5B, SLC22A31,* and *ACE2*) involved in the susceptibility/or protection against severe COVID-19, the complete human genomic landscape of COVID-19 is incomplete. Thus, other approaches, such as candidate gene studies, are required to have a better picture of the SNPs conferring susceptibility to COVID-19 severity and risk of death. For this reason, we length of stay performed the first candidate gene study evaluating the association of SNPs in *PADI2* and *PADI4* with inflammation markers, severity, and death by COVID-19.

The main findings in our study were that: 1) SNPs rs1005753 of *PADI2* and rs874881, rs11203367, and rs11203366 of the *PADI4* gene are associated with susceptibility to severe COVID-19 and death. 2) The haplotypic combination GTACC is associated with susceptibility to severe COVID-19 and death from COVID-19, while the combination GCGTG shows a protective effect against inflammation and death from COVID-19.

SNPs in the *PADI2* and *PADI4* genes associated with susceptibility to severe COVID-19 and death showed a more robust association if combined as haplotypes. PAD2 and PAD4 enzymes play a significant role in the immune system function, regulating the inflammation and triggering innate defenses defense of granulocytes and monocytes against viruses such as SARS-CoV-2 compared with PAD1, PAD3, and PAD6 [16]. PAD2 and PAD4 enzymes participate in the formation of NETs by neutrophils and macrophages via caspase 11-dependent pyroptosis [31], promote neutrophil extravasation and accumulation, and NET formation in lung tissue, contributing to endothelial and lung damage in COVID-19 [8,9,[13], [14], [15]]. Therefore, the citrullination process mediated by PADs enzymes favors defenses against infections during early and acute activation of phagocytosis, neutrophil degranulation, and NET formation. Consequently, early activation of these mechanisms could be vital in containing infectious processes.

SNPs and haplotypes in the *PADI4* gene affect the expression and enzymatic activity of PAD4 [[22], [23], [24], [25]]. Similarly, SNPs in *PADI2* are related to the level of antibodies against citrullinated protein antigens (ACPAs) [32], and the expression of PADI4 (mRNA) is higher in lung biopsies [16] and leukocytes of subjects with severe COVID-19 [17]. Thus, the variability in the expression and function of PADs enzymes secondary to genomic variability could contribute to a differential response against SARS-CoV-2 infection.

The current study found that the genotype and allele frequencies of *PADI2* SNPs rs1005753 and rs2235926 were similar to those previously reported in Mexican [32] and Chinese [26] populations. Similarly, the frequencies for *PADI4* SNPs rs11203366, rs11203367, and rs874881 were similar to those reported in populations from Ukraine [33], Germany [34], Korea [35], China [23], Mexico [24,25,36,37]. However, there were significant differences in the allele distribution of *PADI2* SNPs in our sample compared to other groups referred in the Alpha Project [Supplementary Table 6].

Although the severity and mortality of COVID-19 are related to the presence of comorbidities, pre-existing respiratory diseases, exposure to environmental factors such as smoking [29,38], and even other factors related to the level of economic development of the countries [39,40], genetic variability also contributes to the severity of symptoms and clinical outcomes in patients with COVID-19 [[1], [2], [3], [4]]. Our study demonstrates that some genotypes and haplotypes in *PADI2* and *PADI4* are related to disease severity and death [TT in *PADI2,* GTG and ACC in *PADI4].* The frequency of *PADI2* haplotype was identical to the reported in RA and healthy populations from Mexico [32], and the frequency of *PADI4* haplotypes was similar to the reported in populations from Japan [22], Korea [35], China [23] and, Ukraine [33].

Here, the rs1005753 in PADI2 was individually associated with inflammation and severe COVID-19, whereas rs2235926 was a protective marker for inflammation and death by COVID-19. These findings are plausible since rs1005753 was associated with disease development [41] and with the presence of elevated levels of antibodies to cyclic citrullinated peptides (anti-CCPs), whereas rs2235926 was related to combined seropositivity to ACPAs [32]. The inflammation biomarkers related to SNPs were related to clinical outcomes in previous studies. For example, a high LCRP value was associated with clinical deterioration and the need for IMV in COVID-19 [42]; elevated PLR levels are associated with longer hospitalization time [29], severity, and mortality due to COVID-19 [43]. A meta-analysis reported that patients with COVID-19 with elevated PLR had increased morbidity and mortality from viral infection [44]. Increased platelets and circulating aggregates of platelet-neutrophil-monocytes-T cells are associated with activation of the MAPK pathway and thromboxane-2 generations in COVID-19 [45], promoting platelet hyperreactivity. In addition, during SARS-CoV-2 infection the parallel increase of monocyte tissue factor, fibrinogen and D-dimer levels induces platelet activation in patients requiring IMV and in those who evolved to in-hospital death by COVID-19 [46]. Platelet activation also contributes to the formation of NETs, aggregates and thrombosis observed pulmonary autopsy samples of patients with COVID-19 [9].

Elevated coagulopathy markers such as platelets, D-dimer, prothrombin time, and fibrinogen are also strongly associated with severe COVID-19 [47], particulary has been detected in patients with inflamatory disease that fibrinogen is a sustrate for PAD2 and PAD4 [48], this markers are predictors of COVID-19 severity [49], need for admission to intensive care, requirement of IMV during hospitalization [49,50], and mortality [51]. Consequently, alleles and genotypes associated with negative outcomes might impact mortality and COVID-19 severity through the modulation of the serum levels of such markers, as revealed in our analysis of the association of genotypes and haplotypes with serum biomarkers.

In this study, *PADI4* SNPs were associated with a higher probability of death by COVID-19, and particularly, the SNP rs11203366 was associated with D-dimer levels ≥1.25 μg/mL, which are a marker of severe infection [52]. Carriers of the minor allele of SNPs rs11203366, rs11203367, and rs874881 had CRP levels ≥9.9 mg/dL, an inflammation biomarker associated with poor clinical outcomes in patients with COVID-19 [53]. In this regard, the viral infectious process and inflammation could be aggravated in the early stages of COVID-19 infection, depending on the host's genetic configuration.

The susceptibility for infections attributed to SNPs in *PADI* genes was evaluated in previous studies. In Brazilian patients with septic shock, the SNP rs11203366 of *PADI4* did not affect patient mortality [54]. *PADI4* SNPs rs11203367 and rs874881 were not associated with developing sepsis-induced acute kidney damage, the need for renal replacement therapy, or mortality [55]. The heterozygous rs11203366 genotype of *PADI4* is a protective factor for active tuberculosis in Koreans [56]. In this regard, our findings elucidate the potential role of *PADI2* and *PADI4* SNPs in SARS-CoV-2 infection.

**Fig. 3:**
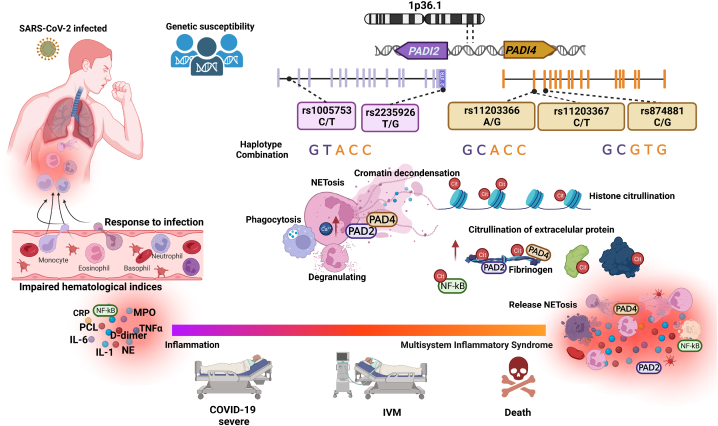
A hypothetical combination of the *PADI2* and *PADI4* polymorphisms contributing to the outcomes of COVID-19. The allele composition in PADs genes could promote a different level of citrullination mediated by PADs enzymes, favoring, or interfering with defense against infections. Allele composition might modulate early and acute activation of phagocytosis, neutrophil degranulation, and NET formation, influencing the control of the infectious processes or worsening the inflammatory response, causing endothelial damage, and progressing to severe COVID-19 or death.

The association of single SNPs and *PADI2* and *PADI4* haplotypes with abnormal markers of inflammation, COVID-19 severity and death might be explained by the higher expression and activity of PAD2 and PAD4 enzymes in monocytes and neutrophils [57,58]. Our results also suggest that SNPs in *PADI2* and *PADI4* play a synergistic role as modifiers of enzymes expression or activity, contributing to the clinical manifestation and outcomes of COVID-19 due to its role in NETosis, cytokine citrullination, platelet activation, and thrombosis (Fig. 3).

Finally, the *in silico* analysis revealed that rs2235926 SNP falls near an intron-exon boundary and had a potential effect on splicing, being it a plausible explanation for the association of this variant with death by COVID-19. However, with current *in silico* tools we cannot explained a mechanism of an association of the rs1005753 SNP with COVID-19 severity. In any case, there is need of further studies for confirm the association of these variants with COVID-19 severity and death in future studies.

This study has limitations, such as lacking a control group of subjects not hospitalized by COVID-19 or with asymptomatic infection. In addition, our study is unicentric, and the genotype distribution may not reflect the reality of all Mexicans and worldwide populations. However, our results provide some perspectives on the influence of PADI2 and PADI4 gene variants on clinical and biochemical COVID-19 outcomes. Furthermore, the literature reviewed also remarks on the importance of analyzing the expression, soluble levels, and enzymatic activity of the isotypes of the PADs enzymes in COVID-19.

## 5 Conclusion

In conclusion, individual SNPs in *PADI2* and *PADI4* were related to COVID-19 severity and death risk. The haplotypic combination GTACC shows susceptibility for the presence of PLR ≥303 and NHL ≥0.83, as well as for severe COVID-19 and COVID-19 death, whereas the haplotypic combination GCGTG shows a protective effect for inflammation and COVID-19 death.

This study provides new insights into the influence of genetic configuration on the susceptibility to severe infection and death by COVID-19. It represents a first step towards recognizing genetic characteristics associated with worse outcomes in respiratory infectious diseases that eventually will lead to the development of personalized medicine focused on preventing high-impact infectious diseases.

## Ethics statement

The study was approved by the local Research Ethics Committee and complied with the Helsinki Declaration (approval number C53-20).

## Funding

None.

## Additional information

No additional information is available for this paper.

## Data availability statement

Data will be a made available on request.

## CRediT authorship contribution statement

**Ilse Adriana Gutiérrez-Pérez:** Writing – review & editing, Writing – original draft, Visualization, Methodology, Investigation, Formal analysis, Data curation. **Ivette Buendía-Roldán:** Methodology. **Oscar Zaragoza-García:** Methodology. **Gloria Pérez-Rubio:** Methodology. **José Rafael Villafan-Bernal:** Methodology. **Leslie Chávez-Galán:** Methodology. **Isela Parra-Rojas:** Methodology. **Rafael de Jesús Hernández-Zenteno:** Methodology. **Ingrid Fricke-Galindo:** Methodology. **Natividad Castro-Alarcón:** Methodology. **Brandon Bautista-Becerril:** Methodology. **Ramcés Falfán-Valencia:** Supervision, Methodology. **Iris Paola Guzmán-Guzmán:** Writing – review & editing, Writing – original draft, Visualization, Validation, Supervision, Methodology, Investigation, Formal analysis, Conceptualization.

## Declaration of competing interest

The authors declare that they have no known competing financial interests or personal relationships that could have appeared to influence the work reported in this paper.
