# Supplementary material for: Association of PADI2 and PADI4 polymorphisms in COVID-19 host severity and non-survival
Source: Heliyon. 2024 Mar 15;10(6):e27997. doi: 10.1016/j.heliyon.2024.e27997 (PMC10958703; doi:10.1016/j.heliyon.2024.e27997)
Supplement: Multimedia component 1 [file mmc1.doc]

**Supplementary material**

| **TABLE S1 Predictor values of biochemical inflammation indices on severity and mortality in COVID-19** | | | | | |
| --- | --- | --- | --- | --- | --- |
| Indices | PaO_2_/FiO_2_ ≤100 | Non-survival | Cut-off value | Sensibility % | Specificity% |
|  | AUC (95% CI), *p* | AUC (95% CI), *p* |  |  |  |
| LCRP | 0.531 (0.479-0.583) 0.225 | 0.602 (0.556-0.648), <0.001 | ≤825 | 65.29 | 40.13 |
| CRP | 0.523 (0.471-0.574) 0.370 | 0.578 (0.532-0.624) 0.001 | ≥9.9 | 65.22 | 51.64 |
| Fibrinogen | 0.554 (0.486-0.622) 0.100 | 0.512 (0.452-0.571) 0.691 | ≥303 | 65.05 | 40.07 |
| D-dimer | 0.640 (0.599-0.681) <0.001 | 0.638 (0.600-0.676), <0.001 | ≥1.25 | 65.82 | 53.60 |
| Procalcitonin | 0.557 (0.509-0.606) 0.020 | 0.630 (0.587-0.673) <0.001 | ≥0.58 | 66.94 | 51.83 |
| Abbreviations: CI, confidence interval; CRP, protein C reactive; LCRP, lymphocyte to CRP ratio.  The AUC was analyzed by ROC curves. *p*-value <0.05 was considered statistically significant. | | | | | |

| **TABLE S2 Association of *PADI2* polymorphisms with hematologic inflammatory markers in COVID-19** | | | | | | | |
| --- | --- | --- | --- | --- | --- | --- | --- |
| **Polymorphisms** | **PLR ≥303**  **OR, 95%CI, *p*** | **NRL ≥11**  **OR, 95%CI, *p*** | **MLR ≥0.58**  **OR, 95%CI, *p*** | **dNLR ≥6.5**  **OR, 95%CI, *p*** | **SII ≥2892**  **OR, 95%CI, *p*** | **SIRI ≥4.29**  **OR, 95%CI, *p*** | **NHL ≥0.83**  **OR, 95%CI, *p*** |
| **rs1005753** |  |  |  |  |  |  |  |
| TG | 1.33 (1.01-1.76) 0.035 | 1.04 (0.79-1.36) 0.767 | 0.90 (0.68-1.19) 0.469 | 0.90 (0.69-1.18) 0.455 | 1.02 (0.78-1.34) 0.845 | 0.99 (0.75-1.31) 0.967 | 1.02 (0.78-1.34) 0.855 |
| GG | 1.32 (0.75-2.38) 0.333 | 0.88 (0.51-1.53) 0.651 | 0.99 (0.68-1.19) 0.469 | 0.66 (0.38-1.15) 0.122 | 0.95 (0.55-1.65) 0.858 | 0.97 (0.55-1.71) 0.913 | 0.94 (0.54-1.63) 0.825 |
| G | 1.23 (0.99-1.52) 0.048 | 0.98 (0.80-1.21) 0.917 | 0.95 (0.77-1.17) 0.643 | 0.86 (0.70-0.05) 0.147 | 1.00 (0.81-1.22) 0.993 | 0.99 (0.80-1.22) 0.946 | 0.99 (0.81-1.22) 0.978 |
| GG+TT | 1.33 (1.02-1.74) 0.028 | 1.01 (0.78-1.30) 0.893 | 0.91 (0.70-1.19) 0.513 | 0.86 (0.67-1.12) 0.264 | 1.01 (0.78-1.31) 0.902 | 0.99 (0.76-1.29) 0.945 | 1.01 (0.78-1.31) 0.921 |
| **rs2235926** |  |  |  |  |  |  |  |
| TC | 0.85 (0.64-1.13) 0.269 | 0.82 (0.62-1.08) 0.156 | 0.73 (0.55-0.97) 0.027 | 0.78 (0.59-1.02) 0.069 | 0.80 (0.60-1.05) 0.105 | 0.84 (0.63-1.11) 0.214 | 0.84 (0.64-1.12) 0.232 |
| CC | 1.34 (0.87-2.10) 0.058 | 1.31 (0.86-2.0) 0.179 | 1.32 (0.85-2.07) 0.091 | 1.27 (0.84-1.92) 0.227 | 1.36(0.89-2.07) 0.129 | 1.17 (0.76-1.81) 0.446 | 1.29 (0.85-1.97) 0.208 |
| C | 1.06 (0.87-1.29) 0.498 | 1.04 (0.80-1.26) 0.613 | 1.01 (0.83-0.22) 0.892 | 1.02 (0.84-1.23) 0.811 | 1.05 (0.87-1.27) 0.574 | 1.01 (0.83-1.22) 0.914 | 1.05 (0.86-1.27) 0.598 |
| TC+CC | 0.94 (0.72-1.24) 0.692 | 0.91 (0.70-1.91) 0.504 | 0.83 (0.63-1.09) 0.179 | 0.87 (0.67-1.13) 0.299 | 0.90 (0.69-1.17) 0.442 | 0.90 (0.69-1.18) 0.416 | 0.93 (0.72-1.21) 0.605 |
| Abbreviations: CI, confidence interval; dNLR, derived neutrophil to lymphocyte ratio; MLR, lymphocyte to monocyte ratio; NHL, neutrophil-to-hemoglobin and lymphocyte; NLR, neutrophil to lymphocyte ratio; OR, odds ratio; PLR, platelet to lymphocyte ratio; SII, systemic immune-inflammation index; SIRI, systemic inflammation response index.  *p*-value <0.05. | | | | | | | |

| **TABLE S3 Association of *PADI2* polymorphisms with inflammatory markers in COVID-19** | | | | | |
| --- | --- | --- | --- | --- | --- |
| **Polymorphisms** | **LCRP ≤825**  **OR, 95%CI, *p*** | **CRP ≥9.9 mg/dL**  **OR, 95%CI, *p*** | **Fibrinogen ≤687 mg/dL**  **OR, 95%CI, *p*** | **D-dimer ≥1.25µg/mL**  **OR, 95%CI, *p*** | **Procalcitonin ≥0.15 mg/dL**  **OR, 95%CI, *p*** |
| **rs1005753** |  |  |  |  |  |
| TG | 0.82 (0.58-1.16) 0.251 | 0.87 (0.62-1.22) 0.412 | 1.10 (0.70-1.72) 0.660 | 1.09 (0.81-1.46) 0.550 | 0.96 (0.69-1.34) 0.831 |
| GG | 0.96 (0.46-2.04) 0.915 | 0.86 (0.42-1.76) 0.655 | 1.49 (0.63-3.72) 0.324 | 0.92( 0.50-1.66) 0.772 | 1.13 (0.57-2.26) 0.703 |
| G | 2.48 (1.86-3.30) <0.001 | 0.90 (0.70-1.17) 0.432 | 2.70 (1.70-4.48) <0.001 | 1.02 (0.81-1.27) 0.856 | 1.01 (0.79-1.29) 0.915 |
| GG+TT | 0.83 (0.60-1.17) 0.287 | 0.87 (0.62-1.20) 0.389 | 1.15 (0.75-1.77) 0.487 | 1.06 (0.80-1.41) 0.652 | 0.98 (0.72-1.35) 0.933 |
| **rs2235926** |  |  |  |  |  |
| TC | 0.89 (0.62-0.29) 0.545 | 1.05 (0.73-1.50) 0.757 | 0.87 (0.54-1.39) 0.553 | 0.71 (0.52-0.96) 0.023 | 0.96 (0.68-1.36) 0.835 |
| CC | 1.15 (0.68-1.95) 0.575 | 0.93 (0.56-1.55) 0.796 | 0.52 (0.26-1.04) 0.044 | 0.83 (0.53-1.29) 0.391 | 0.77 (0.48-1.26) 0.279 |
| C | 1.03 (0.80-1.31) 0.803 | 0.98 (0.77-1.25) 0.915 | 0.75 (0.55-1.03) 0.070 | 0.85 (0.69-1.04) 0.113 | 0.89 (0.71-1.12) 0.331 |
| TC+CC | 0.95 (0.67-1.34) 0782 | 1.02 (0.73-1.43) 0.081 | 0.77 (0.50-1.20) 0.239 | 0.73 (0.55-0.98) 0.0.31 | 0.91 (0.66-1.26) 0.570 |
| Abbreviations: CI, confidence interval: CRP, protein C reactive; LCRP, lymphocyte to CRP ratio; OR, odds ratio.  *p*-value <0.05. | | | | | |

| **TABLE S4 Association of *PADI4* polymorphisms with hematologic inflammatory markers in COVID-19** | | | | | | | |
| --- | --- | --- | --- | --- | --- | --- | --- |
| **Polymorphisms** | **PLR ≥303**  **OR, 95%CI, *p*** | **NRL ≥11**  **OR, 95%CI, *p*** | **MLR ≥0.58**  **OR, 95%CI, *p*** | **dNLR ≥6.5**  **OR, 95%CI, *p*** | **SII ≥2892**  **OR, 95%CI, *p*** | **SIRI ≥4.29**  **OR, 95%CI, *p*** | **NHL ≥0.83**  **OR, 95%CI, *p*** |
| **rs11203666** |  |  |  |  |  |  |  |
| GA | 0.88 (0.64-1.20) 0.427 | 1.05 (0.78-1.43) 0.698 | 0.90 (0.65-1.23) 0.501 | 1.16 (0.86-1.57) 0.287 | 0.91 (0.67-1.23) 0.527 | 0.90 (0.66-1.23) 0.525 | 1.18 (0.87-1.59) 0.259 |
| AA | 1.12 (0.76-1.65) 0.517 | 1.02 (0.71-1.48) 0.870 | 0.85 (0.58-1.24) 0.380 | 0.92 (0.64-1.33) 0.668 | 0.96 (0.66-1.38) 0.835 | 0.88 (0.60-1.28) 0.487 | 1.05 (0.73-1.52) 0.749 |
| A | 1.04 (0.87-1.26) 0.610 | 1.01 (0.84-1.21) 0.843 | 0.91 (0.76-1.10) 0.362 | 0.97 (0.81-1.16) 0.777 | 0.97 (0.81-1.16) 0.785 | 0.93 (0.77-1.12) 0.463 | 1.04 (0.86-1.24) 0.659 |
| GA+AA | 0.95 (0.71-1.27) 0.744 | 1.04 (0.79-1.38) 0.728 | 0.88 (0.66-1.18) 0.399 | 1.08 (0.82-1.43) 0.548 | 0.92 (0.69-1.22) 0.585 | 0.89 (0.67-1.20) 0.457 | 1.14 (0.86-1.51) 0.340 |
| **rs11203667** |  |  |  |  |  |  |  |
| TC | 0.84 (0.61-1.14) 0.258 | 0.99 (0.73-0.34) 0.982 | 0.82 (0.60-1.12) 0.204 | 1.09 (0.81-1.47) 0.550 | 0.89 (0.65-1.20) 0.436 | 0.85 (0.62-1.16) 0.306 | 1.11 (0.82-1.50) 0.466 |
| CC | 1.09 (0.74-1.60) 0.628 | 1.06 (0.73-1.53) 0.730 | 0.84 (0.58-1.23) 0.360 | 0.90 (0.63-1.30) 0.585 | 0.97 (0.67-1.40) 0.884 | 0.87 (0.59-1.26) 0.449 | 1.09 (0.76-1.57) 0.611 |
| C | 1.02 (0.85-1.24) 0.758 | 1.02 (0.85-1.23) 0.745 | 0.90 (0.75-1.09) 0.306 | 0.96 (0.80-1.14) 0.656 | 0.97 (0.81-1.17) 0.812 | 0.92 (0.76-1.11) 0.398 | 1.05 (0.87-1.26) 0.564 |
| TC+CC | 0.91 (0.68-1.22) 0.526 | 1.01 (0.76-1.34) 0.900 | 0.83 (0.61-1.11) 0.196 | 1.02 (0.77-1.35) 0.833 | 0.91 (0.69-1.21) 0.530 | 0.86 (0.64-1.14) 0.293 | 1.10 (0.83-1.46) 0.459 |
| **rs874881** |  |  |  |  |  |  |  |
| GC | 0.91 (0.67-1.23) 0.545 | 0.98 (0.72-1.32) 0.902 | 0.81 (0.59-1.11) 0.179 | 1.09 (0.81-1.47) 0.528 | 0.90 (0.67-1.22) 0.502 | 0.85 (0.62-1.16) 0.298 | 1.09 (0.81-1.47) 0.530 |
| CC | 1.11 (0.75-1.63) 0.577 | 0.99 (0.69-1.44) 0.992 | 0.80 (0.55-1.17) 0.240 | 0.85 (0.59-1.22) 0.361 | 0.90 (0.62-1.31) 0.596 | 0.81 (0.55-1.18) 0.258 | 1.02 (0.71-1.14) 0.874 |
| C | 1.03 (0.86-1.25) 0.674 | 0.99 (0.83-1.19) 0.978 | 0.88 (0.73-1.06) 0.197 | 0.93 (0.78-1.11) 0.447 | 0.94 (0.79-1.13) 0.552 | 0.89 (0.74-1.07) 0.228 | 1.02 (0.85-1.22) 0.807 |
| GC+CC | 0.96 (0.72-1.29) 0.818 | 0.98 (0.74-1.30) 0.924 | 0.81 (0.60-1.08) 0.145 | 1.01 (1.76-1.33) 0.921 | 0.90 (0.68-1.19) 0.479 | 0.84 (0.62-1.12) 0.224 | 1.07 (0.81-1.42) 0.597 |
| Abbreviations: CI, confidence interval; dNLR, derived neutrophil to lymphocyte ratio; MLR, lymphocyte to monocyte ratio; NHL, neutrophil-to-hemoglobin and lymphocyte; NLR, neutrophil to lymphocyte ratio; OR, odds ratio; PLR, platelet to lymphocyte ratio; SII, systemic immune-inflammation index; SIRI, systemic inflammation response index.  *p*-value <0.05. | | | | | | | |

| **TABLE S5 Association of *PADI4* polymorphisms with inflammatory markers in COVID-19** | | | | | |
| --- | --- | --- | --- | --- | --- |
| **Polymorphisms** | **LCRP ≤825**  **OR, 95%CI, *p*** | **CRP ≥9.9 mg/dL**  **OR, 95%CI, *p*** | **Fibrinogen ≤687 mg/dL**  **OR, 95%CI, *p*** | **D-dimer ≥1.25µg/mL**  **OR, 95%CI, *p*** | **Procalcitonin ≥0.15 mg/dL**  **OR, 95%CI, *p*** |
| **rs11203666** |  |  |  |  |  |
| GA | 0.75 (0.51-1.11) 0.143 | 1.00 (0.68-1.46) 0.986 | 1.05 (0.63-1.74) 0.822 | 1.38 (0.99-1.92) 0.046 | 1.13 (0.78-1.64) 0.476 |
| AA | 1.39 (0.84-2.31) 0.165 | 1.75 (1.08-2.48) 0.014 | 0.96 (0.52-1.76) 0.899 | 1.17 (0.78-1.74) 0.415 | 1.07 (0.69-1.67) 0.731 |
| A | 1.13 (0.89-1.43) 0.287 | 1.29 (1.03-1.63) 0.022 | 0.98 (0.73-1.32) 0.920 | 1.10 (0.90-1.34) 0.324 | 1.04 (0.83-1.30) 0.683 |
| GA+AA | 0.90 (0.60-1.30) 0.583 | 1.18 (0.83-1.69) 0.325 | 1.02 (0.63-1.63) 0.913 | 1.31 (0.96-1.79) 0.075 | 1.11 (0.79-1.57) 0.511 |
| **rs11203667** |  |  |  |  |  |
| TC | 0.82 (0.56-1.21) 0.317 | 1.08 (0.74-1.57) 0.667 | 0.96 (0.57-1.59) 0.880 | 1.22 (0.88-1.70) 0.207 | 1.19 (0.82-1.71) 0.326 |
| CC | 1.41 (0.87-2.31) 0.139 | 1.70 (1.06-2.73) 0.018 | 0.93 (0.51-1.70) 0.811 | 1.13 (0.75-1.68) 0.524 | 1.15 (0.73-1.79) 0.514 |
| C | 1.15 (0.91-1.46) 0.217 | 1.30 (1.03-1.63) 0.021 | 0.96 (0.71-1.30) 0.804 | 1.07 (0.88-1.31) 0.451 | 1.08 (0.87-1.35) 0.453 |
| TC+CC | 0.97 (0.67-1.40) 0.891 | 1.24 (0.87-1.77) 0.179 | 0.95 (0.59-1.52) 0.835 | 1.19 (0.87-1.62) 0.239 | 1.17 (0.83-1.65) 0.325 |
| **rs874881** |  |  |  |  |  |
| GC | 0.75 (0.51-1.11) 0.138 | 0.98 (0.67-1.43) 0.925 | 1.04 (0.63-1.73) 0.842 | 1.25 (0.90-1.74) 0.151 | 1.18 (0.82-1.70) 0.341 |
| CC | 1.30 (0.79-2.14) 0.268 | 1.61 (1.00-2.60) 0.036 | 1.00 (0.55-1.85) 0.972 | 1.10 (0.74-1.64) 0.608 | 1.11 (0.71-1.74) 0.604 |
| C | 1.09 (0.86-1.38) 0.438 | 1.24 (0.98-1.56) 0.053 | 1.00 (0.74-1.36) 0.954 | 1.06 (0.84-1.30) 0.497 | 1.07 (0.85-1.33) 0.532 |
| GC+CC | 0.88 (0.61-1.27) 0.504 | 1.14 (0.80-1.62) 0.441 | 1.03 (0.64-1.65) 0.873 | 1.20 (0.88-1.64) 0.207 | 1.16 (0.82-1.63) 0.363 |
| Abbreviations: CI, confidence interval; CRP, protein C reactive; LCRP, lymphocyte to CRP ratio; OR, odds ratio.  *p*-value <0.05. | | | | | |

| **TABLE S6** **Comparison of allele frequencies for polymorphisms in the present study with the Alpha allele frequencies** | | | | | |
| --- | --- | --- | --- | --- | --- |
| **Population** | **Group** | **Sample Size** | **Ref Allele** | **Alt Allele** | ***p* value** |
| **rs1005753_*PADI2*** |  |  |  |  |  |
| Our population | Sub | 1045 | G=0.271 | T=0.729 | *Reference |
| **ALFA Project** |  |  |  |  |  |
| Total | Global | 308002 | G=0.403400 | T=0.596600 | 0.072 |
| South Asian | Sub | 5224 | G=0.2873 | T=0.7127 | 0.875 |
| Other Asian | Sub | 746 | G=0.233 | T=0.767 | 0.624 |
| Other | Sub | 10084 | G=0.41908 | T=0.58092 | 0.037 |
| Latin American 1 | Sub | 1072 | G=0.5121 | T=0.4879 | 0.001 |
| Latin American 2 | Sub | 3160 | G=0.3307 | T=0.6693 | 0.441 |
| European | Sub | 272376 | G=0.398108 | T=0.601892 | 0.072 |
| East Asian | Sub | 3188 | G=0.1048 | T=0.8952 | 0.003 |
| Asian | Sub | 3934 | G=0.1291 | T=0.8709 | 0.021 |
| African Others | Sub | 456 | G=0.754 | T=0.246 | 0.000 |
| African American | Sub | 11696 | G=0.65321 | T=0.34679 | 0.000 |
| African | Sub | 12152 | G=0.65701 | T=0.34299 | 0.000 |
| **rs2235926_*PADI2*** |  |  |  |  |  |
| Our population | Sub | 1045 | T=0.644 | C=0.356 | *Reference |
| **ALFA project** |  |  |  |  |  |
| Total | Global | 149470 | T=0.698682 | C=0.301318 | 0.452 |
| European | Sub | 128666 | T=0.704809 | C=0.295191 | 0.452 |
| African | Sub | 6678 | T=0.6815 | C=0.3185 | 0.654 |
| African Others | Sub | 242 | T=0.752 | C=0.248 | 0.124 |
| African American | Sub | 6436 | T=0.6788 | C=0.3212 | 0.654 |
| Asian | Sub | 630 | T=0.386 | C=0.614 | 0.001 |
| East Asian | Sub | 498 | T=0.404 | C=0.596 | 0.001 |
| Other Asian | Sub | 132 | T=0.318 | C=0.682 | 0.000 |
| Latin American 1 | Sub | 750 | T=0.657 | C=0.343 | 0.882 |
| Latin American 2 | Sub | 6326 | T=0.6473 | C=0.3527 | 0.999 |
| South Asian | Sub | 184 | T=0.592 | C=0.408 | 0.561 |
| **rs11203366_*PADI4*** |  |  |  |  |  |
| Our population | Sub | 1045 | G=0.539 | A=0.461 | *Reference |
| **ALFA project** |  |  |  |  |  |
| Total | Global | 368866 | G=0.421454 | A=0.57854 | 0.119 |
| European | Sub | 312238 | G=0.414572 | A=0.58542 | 0.089 |
| African | Sub | 16524 | G=0.52239 | A=0.47761 | 0.887 |
| African Others | Sub | 594 | G=0.530 | A=0.470 | 0.999 |
| African American | Sub | 15930 | G=0.52210 | A=0.47790 | 0.087 |
| Asian | Sub | 6904 | G=0.4156 | A=0.5844 | 0.119 |
| East Asian | Sub | 4942 | G=0.3942 | A=0.6058 | 0.047 |
| Other Asian | Sub | 1962 | G=0.4694 | A=0.5306 | 0.396 |
| Latin American 1 | Sub | 1420 | G=0.4592 | A=0.5408 | 0.332 |
| Latin American 2 | Sub | 3172 | G=0.4805 | A=0.5195 | 0.480 |
| South Asian | Sub | 5224 | G=0.4663 | A=0.5337 | 0.396 |
| Other | Sub | 23384 | G=0.42345 | A=0.57655 | 0.119 |
| **rs11203367_*PADI4*** |  |  |  |  |  |
| Our population | Sub | 1045 | T=0.541 | C=0.459 | *Reference |
| **ALFA project** |  |  |  |  |  |
| Total | Global | 328360 | T=0.421437 | C=0.578563 | 0.119 |
| African | Sub | 16754 | T=0.49797 | C=0.50203 | 0.671 |
| African American | Sub | 16170 | T=0.49784 | C=0.50216 | 0.671 |
| African Others | Sub | 584 | T=0.502 | C=0.498 | 0.671 |
| Asian | Sub | 6864 | T=0.4151 | C=0.5849 | 0.119 |
| East Asian | Sub | 4886 | T=0.3921 | C=0.6079 | 0.047 |
| European | Sub | 270066 | T=0.414936 | C=0.585064 | 0.089 |
| Latin American 1 | Sub | 1540 | T=0.4455 | C=0.5545 | 0.258 |
| Latin American 2 | Sub | 8820 | T=0.4697 | C=0.5303 | 0.396 |
| Other | Sub | 23936 | T=0.42279 | C=0.57721 | 0.119 |
| Other Asian | Sub | 1978 | T=0.4717 | C=0.5283 | 0.396 |
| South Asian | Sub | 380 | T=0.479 | C=0.521 | 0.480 |
| **rs874881_*PADI4*** |  |  |  |  |  |
| Our population | Sub | 1045 | G=0.549 | C=0.451 | *Reference |
| **ALFA project** |  |  |  |  |  |
| Total | Global | 105512 | G=0.456384 | C=0.543616 | 0.258 |
| European | Sub | 90878 | G=0.45011 | C=0.54989 | 0.203 |
| African | Sub | 2372 | G=0.6632 | C=0.3368 | 0.148 |
| African Others | Sub | 106 | G=0.679 | C=0.321 | 0.181 |
| African American | Sub | 2266 | G=0.6624 | C=0.3376 | 0.148 |
| Asian | Sub | 3236 | G=0.4379 | C=0.5621 | 0.157 |
| East Asian | Sub | 2600 | G=0.4085 | C=0.5915 | 0.066 |
| Other Asian | Sub | 636 | G=0.558 | C=0.442 | 0.999 |
| Latin American 1 | Sub | 672 | G=0.513 | C=0.487 | 0.671 |
| Latin American 2 | Sub | 486 | G=0.658 | C=0.342 | 0.148 |
| South Asian | Sub | 202 | G=0.515 | C=0.485 | 0.777 |
| Other | Sub | 7666 | G=0.4553 | C=0.5447 | 0.258 |
| *Reference population for estimation p value. | | | | | |
